# Effect of Secondary Infections on the Outcomes of Patients with Hematological Malignancies and SARS-CoV-2 Infection: Results from the HM-COV 3.0 Study

**DOI:** 10.3390/v17020274

**Published:** 2025-02-16

**Authors:** Flavia Petrucci, Chiara Pellicano, Francesco Cogliati Dezza, Serena Valeri, Sara Covino, Francesco Iannazzo, Francesca Infante, Antonietta Gigante, Federica Sacco, Agnese Viscido, Alessandra Iacovelli, Edoardo Rosato, Paolo Palange, Claudio Maria Mastroianni, Alessandra Oliva

**Affiliations:** 1 Department of Public Health and Infectious Diseases, Sapienza University of Rome, Piazzale Aldo Moro 5, 00185 Rome, Italy; francesco.cogliatidezza@uniroma1.it (F.C.D.); serena.valeri@uniroma1.it (S.V.); sara.covino@uniroma1.it (S.C.); francesca.infante@uniroma1.it (F.I.); claudio.mastroianni@uniroma1.it (C.M.M.); 2 Department of Translational and Precision Medicine, Sapienza University of Rome, Viale Dell’Università 37, 00185 Rome, Italy; chiara.pellicano@uniroma1.it (C.P.); francesco.iannazzo@uniroma1.it (F.I.); antonietta.gigante@uniroma1.it (A.G.); edoardo.rosato@uniroma1.it (E.R.); 3 Microbiology and Virology Laboratory, Department of Molecular Medicine, Sapienza University of Rome, Piazzale Aldo Moro 5, 00185 Rome, Italy; federica.sacco@uniroma1.it (F.S.); agnese.viscido@uniroma1.it (A.V.); 4 Department of Public Health and Infectious Diseases, Pulmonology Respiratory and Critical Care Unit, Policlinico Umberto I Hospital Rome, 00161 Rome, Italy; alessandra.iacovelli@uniroma1.it (A.I.); paolo.palange@uniroma1.it (P.P.)

**Keywords:** SARS-CoV-2, COVID-19, hematological malignancies, secondary infections, viral reactivation, COVID-19-associated pulmonary aspergillosis (CAPA), mortality

## Abstract

Patients with hematological malignancies (HMs) are at higher risk of severe COVID-19 and secondary infections, which further complicate their outcomes. This study evaluated the impact of secondary infections (SIs) on mortality in hospitalized HM patients with SARS-CoV-2 infection and identified risk factors associated with SIs. We included 217 patients with HMs and COVID-19 admitted to a tertiary hospital in Rome, from April 2020 to September 2022. SIs occurred in 44.2% of patients, with bloodstream infections (42.7%) and respiratory infections (30.5%) being most frequent; among the latter, COVID-19-associated pulmonary aspergillosis (CAPA) was observed in 41.4% of cases. Viral reactivations, predominantly CMV, occurred in 9.2% of patients. The overall mortality rate was 29%, with higher mortality observed in patients with SIs (47.4% vs. 14.7%, *p* < 0.01). Risk factors for SIs included severe COVID-19 (OR = 2.957, *p* < 0.05) and prolonged hospitalization (OR = 1.095, *p* < 0.001). Severe COVID-19 (OR = 8.229, *p* < 0.001), intensive care unit (ICU) admission (OR = 15.232, *p* < 0.001), chronic steroid therapy (OR = 2.803, *p* < 0.05), SIs (OR = 2.892, *p* < 0.05), and viral reactivation (OR = 6.269, *p* < 0.01) were independent predictors of mortality. SIs and viral reactivations are common in patients with HMs and SARS-CoV-2 infection and significantly increase mortality, highlighting the need for timely management and preventive strategies in this vulnerable population.

## 1. Introduction

Since the onset of the COVID-19 pandemic, more than seven million deaths have been reported worldwide, with approximately 200,000 in Italy [1]. Patients with hematological malignancies (HMs) are at a higher risk of severe SARS-CoV-2 infection and tend to experience worse outcomes [2,3]. Mortality rates in this population range from 10% to 40%, and these patients are more likely to require admission to Intensive Care Units (ICUs), experience prolonged viral shedding, and undergo extended hospitalization [4,5,6]. Additionally, this group appears to be particularly prone to the development of secondary infections (SIs), with epidemiological studies reporting a prevalence ranging from 7.7% in an Italian cohort [7] to approximately 19% in a multicenter international study [8]. These infections further complicate their clinical course, especially when multidrug-resistant (MDR) organisms are involved [7,8]. Among secondary infections, particular attention has been drawn to COVID-19-associated pulmonary aspergillosis (CAPA), a complication increasingly recognized in immunocompromised patients and associated with elevated morbidity and mortality rates in those with severe SARS-CoV-2 infection [9,10].

This predisposition to superinfections is consistent with what has been observed in other viral diseases, such as influenza, where secondary bacterial infections contribute significantly to morbidity and mortality [11,12]. Understanding the impact of secondary infections on patients with HMs and COVID-19 is crucial for improving their management and outcomes.

The study aims to assess the impact of secondary infections (SIs) on the outcomes of hospitalized patients with HMs and SARS-CoV-2 infection. Specifically, it will examine mortality rates, the need for admission to ICUs and the duration of the hospital stay. As a secondary objective, the study will also seek to identify the factors that predispose these patients to the development of SIs.

## 2. Materials and Methods

### 2.1. Study Population

This retrospective, single-center study was conducted on patients with HMs and SARS-CoV-2 infection, who were admitted to a tertiary hospital in Rome between April 2020 and September 2022.

Inclusion criteria for the study were: (i) age over 18 years, (ii) confirmed diagnosis of SARS-CoV-2 infection, (iii) diagnosis of a hematological malignancy, and (iv) hospitalization during the study period. Patients under the age of 18 or with non-malignant hematologic diseases were excluded from the analysis.

The cohort was subsequently divided into two groups: those who developed SIs (SI+) and those who did not (SI-), to compare outcomes.

For the purposes of this study, we considered all patients enrolled from the end of January 2022 onward as being affected by the Omicron variant, as Omicron became the dominant strain in Italy by that time, accounting for 99% of cases [13]. For each subject, laboratory and clinical data at hospital admission, as well as treatment data and SARS-CoV-2 RNA time of detection, were collected and recorded anonymously in an electronic database.

### 2.2. Definitions

To confirm SARS-CoV-2 infection, nasopharyngeal swab samples were collected and analyzed by means of molecular (RealStar SARS-CoV2 RT-PCR, Altona Diagnostics, Hamburg, Germany) or antigenic (Liaison, Diasorin, Vercelli, Italy) testings, as appropriate and according to the local protocols. Severity of the SARS-CoV-2 infection was defined according to the WHO classification available at the time of enrolment [14]. The status of the hematological malignancy was defined as remission, refractory/relapsing disease, or yet to be defined, according to the guidelines of the European Society for Medical Oncology [15]. Patients with either a new diagnosis or refractory/relapsing disease were categorized as having active malignancy. Active treatment included receipt of chemotherapy or immunotherapy, or both, in the previous 90 days. Immunotherapy included receipt of monoclonal antibodies (rituximab, daratumumab, and obinutuzumab) and tyrosine kinase inhibitors (imatinib, ibrutinib, ruxolitinib, and venetoclax). The use of corticosteroids within the previous 30 days included therapy with prednisone or its equivalent at a dose > 0.5 mg/kg/day for at least 1 month.

Secondary infection was defined as any infection that developed 48 h after hospital admission, primarily focusing on hospital-acquired infections. Their diagnosis was obtained through microbiological samples collected according to the suspected primary infection site (e.g., blood cultures, urine cultures, sputum, tracheobronchial aspirates, urine, abscesses). Infections were defined according to the CDC/NHSN criteria [16]. Hospital-acquired/ventilator-associated pneumonia (HAP/VAP) was defined in accordance with the CDC/NHSN surveillance definition of healthcare-associated infection for pneumonia with specific criteria [17]. VAP was defined as pneumonia in patients who had a device to assist or control respiration continuously through a tracheostomy or by endotracheal intubation within the 48 h period before the onset of infection.

CAPA was defined according to the recently proposed definitions [18] as well as the practice guidelines [19] using a combination of clinical, radiological, and mycological features of the disease. In case of doubt, a panel discussion was conducted.

### 2.3. Microbiological Analyses

Standard microbiological procedures were performed at hospital admission and/or during hospitalization as requested by the attending physicians, in order to investigate bacterial, viral, and fungal pathogens in the blood, normally sterile fluids, sputum, and other samples, as appropriate. According to routine Hospitals’ Microbiology Laboratory protocol, a bacterial pellet obtained from positive blood cultures was used for bacterial identification by the Matrix-Assisted Laser Desorption Ionization–Time Of Flight Mass Spectrometry (MALDI-TOF MS) system (Bruker Daltonik GmbH, Bremen, Germany). Isolated colonies from other biological samples were identified by the MALDI-TOF MS system (Bruker Daltonik GmbH, Bremen, Germany). Antimicrobial susceptibility testing was performed with the Vitek 2 automated system (bioMérieux, Marcy l’Etoile, France) and Microscan Walkaway (Beckman and Coulter, Brea, CA, USA) system, as appropriate. As for local protocol, screening for MDR bacteria (i.e methicillin-resistant *Staphylococcus aureus* (MRSA) or carbapenem resistant gram-negative bacilli [20]) colonization was systematically performed by means of a rectal/nasal swab or lower respiratory samples, as appropriate.

With regard to CAPA diagnosis, galactomannan (GM), and fungal culture were performed on respiratory samples. Fungal cultures were incubated for 7 days at 30 °C on Sabouraud selective media, whereas a GM test in serum, bronchoalveolar lavage (BAL), and tracheobronchial aspirate (TBA) was performed according to the manufacturer’s instructions (Platelia Aspergillus EIA, Bio-Rad, Milan, Italy).

Viral reactivation was considered when DNA quantification was higher than the limit of detection, supported by clinical signs of infections [21].

In the case of suspected SIs and/or CAPA, the clinical approach was managed together with a dedicated ID physician. In the case of suspected secondary infections, all patients received promptly empiric antibiotic therapy, whereas definitive antibiotic therapy was based on the results of cultures. When feasible, a lung CT scan was performed to detect lesions compatible with invasive pulmonary aspergillosis (IPA) and was analyzed with a dedicated pneumologist and radiologist.

### 2.4. Statistical Analyses

Categorical variables were described through absolute frequencies and percentages; quantitative variables were reported through mean and SD or median with an interquartile range, depending on the normal or non-normal distribution of the data, respectively. The coefficient of kurtosis and skewness was used to evaluate normal or non-normal distribution of data. Differences between qualitative variables were analyzed by means of Chi-square or Fischer’s exact tests, as appropriate, while differences between quantitative variables were assessed by means of t-Student or Mann–Whitney U tests, as appropriate. Univariable analysis was used to identify risk factors and predictors for all-cause in-hospital mortality. Baseline predictors possibly associated with the outcome at univariable comparison and variables considered clinically significant were considered for multivariable logistic regression analysis. Multivariable stepwise logistic regression analysis with an Odds Ratio (OR) and 95% confidence intervals (CIs) was applied to evaluate the association of the following independent variables: age, female sex, Omicron variant, severe COVID-19, chronic steroid therapy, HM remission, eGFR ≤ 45 mL/min, CRP/albumin, admission to ICU, secondary infections, viral reactivation for in-hospital mortality and age, female sex, severe COVID-19, chronic steroid therapy, neutropenia, CRP/albumin, admission to ICU, length of stay for secondary infections development. Variables’ collinearity and interaction were also checked in the final model. *p* values < 0.05 were considered significant. All statistical analyses were performed using STATA^TM^ software, v. 17 (StataCorp, College Station, TX, USA) and Graphpad Prism^TM^ v.8, charts using Microsoft Office^TM^ v. 16 and Graphpad Prism^TM^ v. 8.

### 2.5. Ethics

The study was conducted in accordance with the principles of the Declaration of Helsinki. The protocol was approved by the local Ethics Committee (ID Prot. 109/2020). The clinical and diagnostic management of the patients had already been carried out according to normal clinical practice. Informed consent was waived due to the retrospective nature of the research.

## 3. Results

### 3.1. General Characteristics and Clinical Outcomes

The study included 217 patients with a median age of 69 years [IQR 57–77], of whom 61.7% were male. Omicron infection was present in 51.1% of patients and 64% had been previously vaccinated. Asymptomatic COVID-19 was seen in 18.4% of cases, while 21.2% experienced severe illness. Renal function (eGFR) had a median of 79.7 mL/min, with 15.7% showing eGFR ≤ 45 mL/min. Regarding hematological conditions, the most common were non-Hodgkin lymphoma (38.7%) and chronic lymphocytic leukemia (10.6%). Hematological disease was in remission for 44.7% of patients, while 34.6% were on chronic steroid therapy and 54.4% were on active treatment. ICU admission occurred in 14.3% of patients and the overall mortality rate was 29%; the median hospital stay was 16 days [IQR 9–30]. Other demographic and clinical features of patients are shown in Table 1. 

### 3.2. Secondary Infections and Viral Reactivations

At least one SI was observed in 44.2% of patients, with the most common types being BSI (42.7%) and respiratory tract infections (30.5%). Among respiratory tract infections, HAP/VAP accounted for 58.6%, while CAPA represented 41.4%. These were followed by urinary tract infections, intra-abdominal and other less common infections, including two cases of skin and soft tissue infection, one case of otomastoiditis caused by *Staphylococcus aureus* and one case of mucormycosis. Overall, isolated microorganisms were Gram-positive in 39.2% of cases, Gram-negative in 52.3% and fungi in 8.4%. Specifically, the most frequently isolated pathogens included *Escherichia coli* (13.1%), *Klebsiella pneumoniae* (11.2%), *Acinetobacter baumannii* (11.2%), coagulase-negative *Staphylococci* (10.3%), and *Enterococcus faecium* (8.4%), regardless of the infection type.

When considering specific infection types, we observed the following distribution: BSI was caused mainly by *E. faecium* (19%), followed by *K. pneumoniae*, *E. coli*, *Staphylococcus epidermidis*, and *Staphylococcus haemolyticus* (14.3% each) and *A. baumannii* (12%). With regard to pneumonia, 18,6% were sustained by *A. baumannii* and 14% by *K. pneumoniae*. In 23.2% of pneumonia cases, the pathogen was not identified. Among the other identified pathogens, *Streptococccus pneumoniae* accounted for 7% and *E. coli* and *Enterobacter cloacae* for 4.6% each. Urinary tract infections were caused mainly by *E. coli* (13.3%), *Enterococcus faecalis* (13.3%), *Proteus mirabilis* (13.3%), and *Pseudomonas aeruginosa* (13.3%). Viral reactivation occurred in 9.2% of cases, mostly caused by CMV reactivation (Table 1).

During hospitalization, 18.9% of patients developed colonization by a multidrug-resistant (MDR) microorganism.

### 3.3. Comparison Between Survivors and Non-Survivors

Dividing the patients into two groups based on survival (n = 154, 71%) and death (n = 63, 29%) (Table 2), several key differences were observed. The median age of patients and male gender distribution were not statistically significant (70 vs. 69 years, *p* > 0.05 and 59.1% vs. 68.2%, *p*> 0.05). The median eGFR was lower in non-survivor patients (75.3 mL/min) than in those who survived (81.6 mL/min, *p* > 0.05), although not statistically different. Furthermore, CRP/albumin and D-Dimer levels were significantly elevated in deceased patients (2 vs. 0.7, *p* < 0.001; 1162.5 ng/mL vs. 729.5 ng/mL, *p* < 0.001). A lower percentage of deceased patients (38.1%) had the Omicron variant compared to 56.5% of survivors (*p* < 0.01); instead, the vaccination rate did not show a statistically significant difference between the two groups (57.1% vs. 67.3%, *p* > 0.05). Severe COVID-19 disease was significantly more prevalent among deceased patients (46%) compared to only 11% of survivors (*p* < 0.001). Chronic steroid therapy was more common in the non-survivor group (50%) compared to the survivor group (28.8%, *p* < 0.01). Additionally, 41.3% of those who died were admitted to the ICU, compared to only 3.2% of survivors (*p* < 0.001). Patients who died had a longer median hospital stay of 20 days, in contrast to 16 days for survivors; however, this difference was not statistically significant (*p* > 0.05). The prevalence of MDR colonization was significantly higher among deceased patients (36.5%) compared to survivors (11.7%, *p* < 0.001) and, similarly, non-survivors had a higher percentage of SIs (71.4% vs. 32.4%, *p* < 0.001) and viral reactivations (19% vs. 5.2%, *p* < 0.001) (Table 2).

In the multivariable logistic regression analysis, mortality was significantly associated with severe SARS-CoV-2 disease [OR = 8.229 (95% CI: 2.913–23.249); *p* < 0.001], chronic steroid therapy [OR = 2.803 (95% CI: 1.003–7.830); *p* < 0.05], admission to the ICU [OR = 15.232 (95% CI: 3.838–60.452); *p* < 0.001], eGFR ≤ 45 mL/min [OR = 11.338 (95% CI: 3.273–39.278); *p* < 0.001], secondary infections [OR = 2.892 (95% CI: 1.093–7.649); *p* < 0.05], and viral reactivations [OR = 6.269 (95% CI: 1.734–55.669); *p* < 0.01] (Table 3).

### 3.4. Comparison Between Patients With and Without Secondary Infections

Overall, a total of 96 (44.2%) patients developed SIs. Severe COVID-19 disease was more common among patients with SIs (30.2%) than in those without (14%, *p* < 0.01). Patients with SIs were more frequently on chronic steroid therapy (48.4% vs. 24.2%, *p* < 0.001) and were more often neutropenic (15.6% vs. 6.7%, *p* < 0.05). CRP/albumin levels were significantly higher in patients with secondary infections (1.7) compared to those without (0.7, *p* < 0.001). Additionally, 71% of those who developed a SI were admitted to the ICU, compared to only 29% of those who did not (*p* < 0.001) and MDR colonization was markedly more frequent in patients with a SI (35.4%) than in those without (5.8%, *p* < 0.001). The mortality rate was significantly higher among patients with SIs (47.4%) compared to those without (14.7%, *p* < 0.01). Likewise, patients with SIs had a significantly longer median hospital stay of 30 days, compared to just 11 days for those without SIs (*p* < 0.001) (Table 2).

Examining the risk factors for SIs, the multivariable logistic regression analysis revealed that secondary infections development was significantly associated with severe SARS-CoV-2 disease [OR = 2.957 (95% CI: 1.258–6.950); *p* < 0.05], length of stay [OR = 1.095 (95% CI: 1.056–1.136); *p* < 0.001], chronic steroid therapy [OR = 2.902 (95% CI: 1.421–5.925); *p* < 0.01] and neutropenia [OR = 3.280 (95% CI: 1.067–10.087); *p* < 0.05] (Table 3).

## 4. Discussion

The present study describes the clinical characteristics and outcomes of hospitalized patients with HMs and SARS-CoV-2 infection, along with the evaluation of predictors of SIs development and the impact of SIs on mortality. We found that SIs were an independent risk factor for mortality in this high-risk population and that the severity of SARS-CoV-2 disease as well as immunosuppression (expressed by chronic steroid therapy and neutropenia) were predictors of SIs.

It is well known that the HM population presents a higher rate of complications and worse outcome and specific kinetics of respiratory worsening [2,3,4,5,6], and this was confirmed by the high mortality rate found also in the present study (29%). Drivers of worse outcome in the present study were not only the severity of the SARS CoV2 infection or a chronic steroid therapy but also SIs (including CAPA) and viral reactivation. Secondary infections in COVID-19 patients are reported in the literature with a prevalence of around 22% [22,23], especially in ICUs, where they represented an independent predictor of mortality [24].

So far, limited data are available about the impact of SIs in HM patients hospitalized for COVID-19 disease; our study reported a 44.2% rate of secondary infections, higher than in similar studies, where rates vary between 7% and 19% [7,8].

According to the literature, we observed a numerically lower mortality rate in patients infected with Omicron VOCs (38.1% deceased patients vs. 56.5% survived, *p* < 0.01), which, however, did not result an independent predictor of mortality. Although in the general population, the Omicron variant is associated with a lower mortality rate [25,26], studies in hematology patients have shown only a slight reduction in mortality compared to previous VOCs. Unlike immunocompetent individuals, infection with Omicron in HM patients remains associated with significant mortality among hospitalized individuals [27].

The occurrence of secondary infections in 44.2% of patients further highlights the challenges posed by compromised immune function in individuals with hematological diseases. Bloodstream infections were the most frequent secondary infections, with *E. faecium*, *E. coli*, *S. epidermidis*, and *A. baumannii* being the predominant causative agents. BSI had a similar distribution of Gram-negative and Gram-positive organisms, and the specific type of isolates did not appear to influence survival. Gram-positive bacteria may be partially explained by the frequent use of central venous catheters in critically ill and frail patients, while the high prevalence of Gram-negative bacteria is consistent with recent epidemiological data reported in HM patients [28]. Pneumonia and urinary tract infections also featured prominently, while intra-abdominal infections were primarily attributed to *Clostridium difficile*.

We found that CAPA occurred in 5.52% of patients and represented 41.4% of the respiratory infection in our cohort. There is a growing interest in coronavirus-associated pulmonary aspergillosis (CAPA) and its association with critical illness [9,29]. The prevalence of CAPA in our study (5.52%) falls within the range widely reported in the literature (5–30%) [10]. However, it is interesting to note that the variation between studies may reflect differences in diagnostic criteria, patient characteristics (e.g., the underlying hematological disease) or limitations in diagnostic investigations during the pandemic. Specifically, our incidence may have been influenced by difficulties in performing invasive diagnostic procedures, such as bronchoscopy, due to COVID-19 containment measures and/or patients’ clinical conditions. However, although data about HM patients are few, CAPA represents a significant complication and risk factor for mortality even in immunocompetent patients [29]. Therefore, recognizing CAPA symptoms and raising a diagnostic suspicion can be challenging, but it is necessary to prevent fatal outcomes, especially in high-risk patients, such as those with an HM, allowing for the prompt initiation of prophylaxis, if necessary.

One interesting aspect of the study is the finding that viral reactivation, which affected 9.2% of patients and, as expected, was predominantly linked to CMV reactivation, was a predictor itself of mortality. While the literature reports data on viral reactivation in critically ill COVID-19 patients [30,31], few studies have investigated the impact of these reactivations on patient mortality, particularly in those with hematologic malignancies. This highlights the complex interplay between immune suppression, underlying hematological diseases, and viral reactivations, necessitating vigilant monitoring and tailored preventive strategies since there is a direct impact on mortality. 

As expected, patients with SIs exhibited a significantly prolonged median length of stay compared to those without (30 vs. 11 days), underscoring the burden of secondary infections on healthcare resources and patient well-being.

In our cohort, we found an overall mortality of 29%: the higher mortality rate observed in our study, compared to other reports, could be attributed to several factors. All patients included in our cohort required hospitalization, which likely contributed to the overall high mortality. Additionally, the median age of our patients was relatively high, which may have further increased the risk of adverse outcomes. More than half of the patients were not infected with the Omicron variant, and many were undergoing active treatment for their underlying hematological malignancy, which could have made them more vulnerable to severe COVID-19 and associated complications, such as SIs, which we found to be independently associated with mortality.

SIs significantly contribute to mortality in HM patients through various mechanisms. Immune suppression, either due to underlying malignancies, SARS-CoV-2 infection itself or treatments received for the underlying malignancy, predisposes these patients to a higher risk of bacterial, fungal, and viral infections. Once established, SIs exacerbate systemic inflammation and immune dysregulation, possibly leading to septic syndrome and multiorgan failure, both of which are major causes of mortality in this population. Additionally, bloodstream infections, which were among the most frequent SIs in our cohort, are known to have particularly high mortality rates in critically ill patients due to their rapid progression and difficulty of management. Similarly, viral reactivation, particularly CMV, may act as a predictor of mortality by further compromising the immune response. CMV reactivation is associated with a pro-inflammatory state, cytokine storm and end-organ damage, which can exacerbate the already fragile clinical condition of HM patients. The interplay between viral reactivation and immune suppression creates a vicious cycle, increasing the likelihood of co-infections and further patients’ deterioration.

This study inevitably presents some limitations, primarily due to its retrospective design and single-center nature. The small number of patients did not allow for stratification by hematological pathology or type of SIs, and, consequently, the potential impact of these differences on disease outcomes remains unclear. Furthermore, the inclusion of patients from the early pandemic phase, when therapeutic guidelines were not yet fully established and broad-spectrum antibiotics were often inappropriately used, may have influenced the incidence of SIs observed in our cohort. In line with this, we included patients from different pandemic phases (e.g., the first wave and the Omicron phase), potentially introducing additional treatment strategy variability. To mitigate these limitations in future research, we plan to conduct larger, multicenter studies with improved data collection and more comprehensive patient stratification. Addressing these biases and expanding the scope of the research will enable a more accurate understanding of the factors that contribute to poor outcomes in HM patients with SARS-CoV-2 infection. Nevertheless, our findings reinforce the fragility of this patient population, which remains highly susceptible to SIs, that further worsen the prognosis. Additional studies are needed to better identify risk factors and refine strategies to prevent unfavorable outcomes in this vulnerable group.

## 5. Conclusions

In conclusion, this study sheds light on the complex clinical landscape faced by inpatients with hematological diseases, particularly in the context of SARS-CoV2 infection. The high prevalence of secondary infections including viral reactivation and their association with increased mortality highlights the need for tailored management approaches that consider the unique challenges posed by both the underlying hematological conditions and the infectious agents. By identifying key risk factors and their implications, this research paves the way for improved patient care and informs future interventions aimed at reducing the morbidity and mortality in this vulnerable population. Further studies are warranted to explore specific preventive and therapeutic strategies targeting secondary infections in this cohort.

## Figures and Tables

**Table 1:** Demographic and clinical features of 217 patients enrolled. All results are reported as median and interquartile range (IQR) or absolute frequency and percentage.

Variable	Value
Age, median (IQR)	69 (57–77)
Male/Female, n (%)	134 (61.7)/83 (38.2)
Systemic arterial hypertension, n (%)	96 (44)
Cardiovascular disease, n (%)	37 (17)
Diabetes mellitus, n (%)	29 (13.4)
COPD, n (%)	20 (9.2)
Omicron variant, n (%)	111 (51.1)
Vaccination, n (%)	139 (64)
Asymptomatic COVID-19, n (%)	40 (18.4)
Severe COVID-19, n (%)	46 (21.2)
eGFR, ml/min, median (IQR)	79.7 (58.8–96.5)
eGFR ≤ 45 mL/min, n (%)	34 (15.7)
Neutropenia, n (%)	23 (10.6)
Hematological disease, n (%)	
ALL	10 (4.6)
AML	22 (10.1)
CML	7 (3.2)
CLL	23 (10.6)
PLL	2 (0.9)
PL	1 (0.5)
MM	23 (10.6)
MS	17 (7.8)
Myelofibrosis	9 (4.1)
NHL	84 (38.7)
HL	8 (3.7)
Others	11 (5.1)
Remission of hematological disease, n (%)	97 (44.7)
Chronic steroid therapy, n (%)	75 (34.6)
Active treatment, n (%)	118 (54.4)
MDR colonization, n (%)	41 (18.9)
Secondary infection, n (%)	96 (44.2)
BSI °	41 (42.7)
Pneumonia *	29 (30.5)
UTI	15 (14.7)
Intra-abdominal	6 (6.3)
Others ^†^	4 (4.2)
Viral reactivation ^§^, n (%)	20 (9.2)
Exitus	63 (29)
Length of stay, days	16 (9–30)
Admission to ICU, n (%)	31 (14.3)

° *E. faecium* (19%), *S. epidermidis* (14.3%), *K. pneumoniae* (14.3%), *E. coli* (14.3%), *S. haemolyticus* (14.3%), and *A. baumannii* (12%). * HAP/VAP 17 (58.6%), CAPA 12 (41.4%), HAP/VAP mainly caused by *A. baumannii* (18.6%) and *K. pneumoniae* (14%). ^†^ 2 patients with skin and soft tissue infection, 1 patient with *S. aureus* otomastoiditis, and 1 patient with mucormycosis. ^§^ CMV 15 (78.9%), other Herpesviruses 4 (21%). ALL: acute lymphocytic leukaemia, AML: acute myeloid leukaemia, CML: chronic myeloid leukaemia, CLL: chronic lymphocytic leukaemia, PLL: prolymphocytic leukemia, PL: plasma cell leukemia, MM: multiple myeloma; MS: myelodysplastic syndrome, NHL: non-Hodgkin lymphoma, HL: Hodgkin lymphoma, ICU: Intensive Care Unit, eGFR: estimated glomerular filtration rate, CRP: C-reactive protein, COPD: chronic obstructive pulmonary disease. BSI: bloodstream infections, MDR: multidrug-resistant, UTI: urinary tract infections, HAP: hospital-acquired pneumonia, VAP: ventilator-associated pneumonia, CAPA: COVID-19 associated pulmonary aspergillosis.

**Table 2:** Comparative analysis of 217 patients enrolled, grouped according to exitus or development of secondary infections. All results are reported as median and interquartile range (IQR) or absolute frequency and percentage. Mann–Whitney U tests were used to compare variables where medians are reported and a Chi-square or Fisher’s exact test was used to compare variables where frequencies are reported.

Variables	Exitus (n = 63)	No Exitus (n = 154)	*p*	Secondary Infection (n = 96)	No Secondary Infection (n = 121)	*p*
Age, median (IQR)	70 (61.5–79)	69 (55–76.5)	>0.05	69.5 (59.7–78)	69 (53–77)	>0.05
Gender male, n (%)	91 (59.1)	43 (68.2)	>0.05	65 (67.7)	69 (57)	>0.05
Systemic arterial hypertension, n (%)	29 (46)	67 (43.5)	>0.05	41 (42.7)	55 (45.4)	>0.05
Cardiovascular diseases, n (%)	9 (14.3)	28 (18.2)	>0.05	16 (16.7)	21 (17.3)	>0.05
Diabetes mellitus, n (%)	6 (9.5)	23 (14.9)	>0.05	11 (11.4)	18 (14.9)	>0.05
COPD, n (%)	8 (12.7)	12 (7.8)	>0.05	9 (9.4)	11 (9.1)	>0.05
Omicron variant, n (%)	24 (38.1)	87 (56.5)	<0.01	54 (56.2)	57 (47.1)	>0.05
Vaccination, n (%)	36 (57.1)	103 (67.3)	>0.05	66 (68.7)	73 (60.8)	>0.05
Asymptomatic COVID-19, n (%)	8 (12.7)	32 (20.8)	>0.05	21 (21.9)	19 (15.7)	>0.05
Severe COVID-19, n (%)	29 (46)	17 (11)	<0.001	29 (30.2)	17 (14)	<0.01
eGFR, ml/min, median (IQR)	75.3 (49.9–95.2)	81.6 (61.9–96.9)	>0.05	77.7 (57.2–94.7)	81.6 (61.2–100.6)	>0.05
eGFR ≤ 45 mL/min, n (%)	20 (31.7)	14 (9.1)	<0.001	19 (19.8)	15 (12.4)	>0.05
CRP/albumin, median (IQR)	2 (0.9–4.9)	0.7 (0.3–1.9)	<0.001	1.7 (0.6–3.6)	0.7 (0.2–1.6)	<0.001
D-Dimer, ng/mL, median (IQR)	1162.5 (798.5–2473)	729.5 (415–1554)	<0.001	1156 (632.5–2665)	736 (415–1424.2)	<0.01
Neutropenia, n (%)	10 (15.9)	13 (8.5)	>0.05	15 (15.6)	8 (6.7)	<0.05
Hematological disease, n (%)	1 (1.6)	9 (5.8)	>0.05	5 (5.2)	5 (4.1)	>0.05
ALL	8 (12.7)	14 (9.1)	>0.05	13 (13.5)	9 (7.4)	>0.05
AML	1 (1.6)	6 (3.9)	>0.05	2 (2.1)	5 (4.1)	>0.05
CML	7 (11.1)	16 (10.4)	>0.05	11 (11.4)	12 (9.9)	>0.05
CLL	0 (0)	2 (1.3)	>0.05	1 (1)	1 (0.8)	>0.05
PLL	1 (1.6)	0 (0)	>0.05	0 (0)	1 (0.8)	>0.05
PL	7 (11.1)	16 (10.4)	>0.05	9 (9.4)	14 (11.6)	>0.05
MM	3 (4.7)	14 (9.1)	>0.05	9 (9.4)	8 (6.6)	>0.05
MS	2 (3.2)	7 (4.5)	>0.05	2 (2.1)	7 (5.8)	>0.05
Myelofibrosis	30 (47.6)	54 (35.1)	>0.05	37 (38.5)	47 (38.8)	>0.05
NHL	3 (4.8)	5 (3.3)	>0.05	3 (3.1)	5 (4.1)	>0.05
HL	0 (0)	2 (1.3)	>0.05	1 (1)	1 (0.8)	>0.05
Others	0 (0)	1 (0.7)	>0.05	1 (1.3)	0 (0)	>0.05
Remission of hematological disease, n (%)	21 (33.3)	76 (49.3)	<0.05	41 (42.7)	56 (46.3)	>0.05
Chronic steroid therapy, n (%)	31 (50)	44 (28.8)	<0.01	46 (48.4)	29 (24.2)	<0.001
Active treatment, n (%)	32 (50.8)	86 (55.8)	>0.05	52 (54.2)	66 (54.5)	>0.05
Length of stay, days median (IQR)	20 (10.5–30)	16 (9–30)	>0.05	30 (14.7–30)	11 (7–21)	<0.001
Admission to ICU, n (%)	26 (41.3)	5 (3.2)	<0.001	22 (71)	9 (29)	<0.001
MDR colonization, n (%)	23 (36.5)	18 (11.7)	<0.001	34 (35.4)	7 (5.8)	<0.001
Secondary infectious disease, n (%)	45 (71.4)	50 (32.4)	<0.001	NA	NA	NA
Bloodstream infection	26 (41.3)	15 (9.7)				
Respiratory infection *	10 (15.9)	19 (12.3)				
Urinary tract infection	4 (6.3)	11 (7.1)				
Intra-abdominal	4 (6.3)	2 (1.3)				
Others	0 (0)	3 (1.9)				
Viral reactivation, n (%)	12 (19)	8 (5.2)	<0.001	NA	NA	NA
Exitus, n (%)	NA	NA	NA	45 (47.36)	18 (14.7)	<0.01

* including HAP/VAP and CAPA. ALL: acute lymphocytic leukemia, AML: acute myeloid leukemia, CML: chronic myeloid leukemia, CLL: chronic lymphocytic leukemia, PLL: prolymphocytic leukemia, PL: plasma cell leukemia, MM: multiple myeloma; MS: myelodysplastic syndrome, NHL: non-Hodgkin lymphoma, HL: Hodgkin lymphoma, ICU: Intensive Care Unit, eGFR: estimated glomerular filtration rate, CRP: C-reactive protein, COPD: chronic obstructive pulmonary disease, MDR: multidrug-resistant, UTI: urinary tract infection, HAP: hospital-acquired pneumonia, VAP: ventilator-associated pneumonia, CAPA: COVID-19-associated pulmonary aspergillosis.

**Table 3:** Multivariable logistic regression analysis with odds (OR) and 95% confidence interval (CI) for exitus and secondary infections.

**Dependent Variable: Exitus**		
**Independent Variables**	**OR (95% CI)**	***p***
Age	1.010 (0.977; 1.044)	>0.05
Female Sex	0.901 (0.325; 2.494)	>0.05
Omicron	0.707 (0.260; 1.917)	>0.05
Severe COVID-19	8.229 (2.913; 23.249)	<0.001
Chronic steroid therapy	2.803 (1.003; 7.830)	<0.05
Remission	0.488 (0.183; 1.306)	>0.05
eGFR ≤ 45 mL/min	11.338 (3.273; 39.278)	<0.001
CRP/albumin	1 (0.993; 1.006)	>0.05
Admission to ICU	15.232 (3.838; 60.452)	<0.001
Secondary infections	2.892 (1.093; 7.649)	<0.05
Viral reactivation	6.269 (1.734; 22.669)	<0.01
**Dependent Variable: Secondary Infections**		
**Variables**	**OR (95% CI)**	***p***
Age	1.007 (0.983; 1.032)	>0.05
Female sex	0.697 (0.331; 1.468)	>0.05
Severe COVID-19	2.957 (1.258; 6.950)	<0.05
Chronic steroid therapy	2.902 (1.421; 5.925)	<0.01
Neutropenia	3.280 (1.067; 10.087)	<0.05
CRP/albumin	0.999 (0.989; 1.009)	>0.05
Admission to ICU	1.221 (0.444; 3.359)	>0.05
Length of stay	1.095 (1.056; 1.136)	<0.001

ICU: Intensive Care Unit, eGFR: estimated glomerular filtration rate, CRP: C-reactive protein.

## Data Availability

All data relevant to the study are included in the article.
